# Assessing Information Provided by ChatGPT: Heart Failure Versus Patent Ductus Arteriosus

**DOI:** 10.7759/cureus.86365

**Published:** 2025-06-19

**Authors:** Meghana Bhupathi, Jaza Mehweish Kareem, Anjali Mediboina, Keerthana Janapareddy

**Affiliations:** 1 Pediatric Medicine, Alluri Sitarama Raju Academy of Medical Sciences, Eluru, IND; 2 Data Quality, West Anaheim Medical Center, Anaheim, USA; 3 Community Medicine, Alluri Sitarama Raju Academy of Medical Sciences, Eluru, IND; 4 Internal Medicine, Gayatri Vidya Parishad Institute of Health Care and Medical Technology, Visakhapatnam, IND

**Keywords:** ai, cardiac conditions, chatgpt, heart failure, medicine, patent ductus arteriosus, pda

## Abstract

Introduction

The study aims to provide insights regarding the effectiveness of ChatGPT in providing accurate, complete, and understandable information related to heart failure (HF) and patent ductus arteriosus (PDA). The study aimed to identify whether the abundance of online resources and patient inquiries leads to ChatGPT providing more accurate, complete, and understandable responses for HF compared to the less common PDA.

Methodology

Ten open-ended questions related to HF and 11 questions related to PDA were formed, and answers generated in ChatGPT 3.5 were assessed for accuracy via a 6-point Likert scale and for completeness via a 3-point Likert scale. Understandability was assessed using the Patient Education Materials Assessment Tool for Printable Materials (PEMAT-P). Data analysis and visualization was conducted using R Software version 4.3.1. Statistical significance between accuracy and completeness for both the conditions was determined using Spearman’s R and Mann-Whitney U tests.

Results

Relevant answers were generated for all questions on the first attempt. For HF-related answers, the mean accuracy score was 5.4 (SD=0.7) and the mean completeness score was 2.3 (SD=0.7), with a median understandability of 83.75% (IQR=77.8%-94.5%). Spearman's correlation coefficient (rs) for HF was 0.44854 (p=0.19353). For PDA, the mean accuracy was 5.09 (SD=0.7), completeness was 2.27 (SD=0.5), and median understandability was 94.5% (IQR=88.9%-100%). Spearman's correlation coefficient for PDA was 0.21409 (p=0.52731). Furthermore, the Mann-Whitney U-test revealed no significant differences in accuracy (p=0.35758) or completeness (p=0.88866) between the two conditions.

Conclusions

While the accuracy and completeness of answers related to HF were slightly higher compared to those for PDA, the understandability of PDA responses was notably better. There is a need for refining artificial intelligence-driven language models for medical information dissemination.

## Introduction

Artificial intelligence (AI) is being increasingly used in various industries for its ability to generate human-like text based on the input it receives [1-3]. In the realm of healthcare specifically, ChatGPT offers promising applications by providing instant access to diagnostic suggestions, treatment options, and explanations of medical conditions. For patients, ChatGPT can also serve as a readily accessible source of information, helping them understand their symptoms, conditions, and potential treatments. This immediacy and accessibility are particularly beneficial in an era where digital health information is sought by patients for quick and reliable answers to their medical queries [4,5]. Despite these advantages, the efficacy of AI-driven chatbots in delivering accurate and comprehensive medical information remains underexplored.

Heart failure (HF) is a common cardiac condition characterized by the heart's inability to pump blood efficiently, leading to symptoms such as shortness of breath, fatigue, and fluid retention [6]. The etiology of HF is multifactorial, encompassing coronary artery disease, hypertension, valvular heart disease, and cardiomyopathies [6]. It is a prevalent condition with significant morbidity and mortality, making it a frequent subject of patient inquiries and healthcare consultations. In contrast, patent ductus arteriosus (PDA) is a less common congenital heart defect in which the ductus arteriosus, a blood vessel that connects the pulmonary artery to the descending aorta in the fetal heart, fails to close after birth [7]. This condition can lead to abnormal blood flow between the aorta and pulmonary artery, potentially causing respiratory distress, HF, and increased risk of endocarditis [7]. PDA is primarily observed in premature infants, and its management may involve pharmacological interventions, catheter-based procedures, or surgery [7].

Given the widespread availability of information on common conditions such as HF compared to less common conditions such as PDA, this study aims to investigate the efficacy of ChatGPT in providing medical information for both conditions. We hypothesize that ChatGPT, due to the abundance of online resources and frequent patient inquiries, will provide more accurate, complete, and understandable responses for HF compared to PDA [2]. A similar finding was noted by Agrawal et al., who attributed ChatGPT's better performance in general medicine and mental health queries to the higher volume of publicly available training data in these areas [8]. Studies such as those by Mehnen et al. and Huh further observed that ChatGPT's diagnostic performance tended to decline with the increasing disease complexity and/or rarity and that ChatGPT performed best on common conditions [9,10].

Thus, by comparing two conditions, HF and PDA, this study seeks to assess the effectiveness and utility of ChatGPT in addressing varying medical needs and conditions. This research aims to provide insights into the potential of AI chatbots to serve as reliable sources of medical information and identify areas for improvement in AI-driven patient education tools.

Aims and objectives

The objective of this study is to evaluate the accuracy, completeness, and understandability of ChatGPT-generated responses for HF and PDA to assess its reliability as a source of medical information for both common and rare cardiac conditions.

## Materials and methods

Study design and setting

Two sets of 10 open-ended questions related to HF and 11 questions related to PDA were formed based on FAQs from the American Heart Association (AHA) website. Answers were generated in ChatGPT 3.5 by a single researcher (A.M.) on February 18, 2024, to ensure consistency. The ChatGPT-generated answers were subsequently assessed by two independent evaluators by referring to the information provided by the AHA. Any disparities were further cross-checked against the American College of Cardiology (ACC) guidelines.

Scoring criteria and study tools

The generated information was evaluated for three criteria: accuracy, completeness, and understandability. Accuracy and completeness were assessed using a scale set by Johnson et al. and used in studies such as those by Goodman et al. Accuracy was assessed using a six-point Likert scale ranging from 1 to 6 for accuracy (Table 1) [11,12].

**Table 1:** Johnson et al.'s accuracy scale Source: [11]

Accuracy Score	Interpretation
1	Completely incorrect
2	More incorrect than correct
3	Approximately equal correct and incorrect
4	More correct than incorrect
5	Nearly all correct
6	Correct

The three-point scale for completeness is shown in Table 2 [11].

**Table 2:** Johnson et al.'s completeness scale Source: [11]

Completeness Score	Interpretation
1	Incomplete, addresses some aspects of the question, but significant parts are missing or incomplete
2	Adequate, addresses all aspects of the question, and provides the minimum amount of information required to be considered complete
3	Comprehensive, addresses all aspects of the question, and provides additional information or context beyond what was expected

Understandability was assessed using the Patient Education Materials Assessment Tool for Printable Materials (PEMAT-P) (Table 3). The PEMAT-P tool was designed to evaluate the quality of patient education materials and consists of two main components: understandability and actionability [13]. In this study, we focused exclusively on the 17 understandability items for printable materials due to the primary aim of determining how well ChatGPT's responses could be comprehended by patients. Each item is rated on a binary scale (agree/disagree), where a score of "1" indicates that the material meets the criterion and a score of "0" indicates that it does not.

**Table 3:** Patient Education Materials Assessment Tool for Printable Materials (PEMAT-P) Source: [13] *A very short print material is defined as a material with two or fewer paragraphs and no more than one page in length. **Items 13 and 14 are for audio-visual materials (PEMAT-A/V) and hence are omitted Understandability Score = Total Points / Total Possible Points x 100

Item #	Item	Response Options
Topic: Content		
1	The material makes its purpose completely evident.	Disagree=0, agree=1
2	The material does not include information or content that distracts from its purpose.	Disagree=0, agree=1
Topic: Word Choice and Style		
3	The material uses common, everyday language.	Disagree=0, agree=1
4	Medical terms are used only to familiarize audience with the terms. When used, medical terms are defined.	Disagree=0, agree=1
5	The material uses the active voice.	Disagree=0, agree=1
Topic: Use of Numbers		
6	Numbers appearing in the material are clear and easy to understand.	Disagree=0, agree=1, no numbers=N/A
7	The material does not expect the user to perform calculations.	Disagree=0, agree=1
Topic: Organization		
8	The material breaks or “chunks” information into short sections.	Disagree=0, agree=1, very short material*=N/A
9	The material’s sections have informative headers.	Disagree=0, agree=1, very short material*=N/A
10	The material presents information in a logical sequence.	Disagree=0, agree=1
11	The material provides a summary.	Disagree=0, agree=1, very short material*=N/A
Topic: Layout and Design		
12	The material uses visual cues (e.g., arrows, boxes, bullets, bold, larger font, highlighting) to draw attention to key points.	Disagree=0, agree=1, video=N/A
Topic: Use of Visual Aids		
15**	The material uses visual aids whenever they could make content more easily understood (e.g., illustration of healthy portion size).	Disagree=0, agree=1
16	The material’s visual aids reinforce rather than distract from the content.	Disagree=0, agree=1, no visual aids=N/A
17	The material’s visual aids have clear titles or captions.	Disagree=0, agree=1, no visual aids=N/A
18	The material uses illustrations and photographs that are clear and uncluttered.	Disagree=0, agree=1, no visual aids=N/A
19	The material uses simple tables with short and clear row and column headings.	Disagree=0, agree=1, no visual aids=N/A

The scores for all items are summed and then multiplied by 100 to obtain a percentage to reflect the overall understandability of the material. A higher percentage indicates greater understandability, and the developers have set the cutoff value for understandability at 70%.

Data analysis

Answers were generated in ChatGPT 3.5 by a single researcher (A.M.), after which two evaluators first independently and blindly evaluated and scored the answers provided by ChatGPT 3.5. The assigned scores were then compared, and any discrepancies were meticulously reviewed and discussed by the evaluators to reach a consensus. In cases of differing scores, consensus was achieved through thorough discussion and re-evaluation of the responses. This process aimed to eliminate potential bias and subjectivity in the scoring. The final consensus scores were used for analysis.

Data were then exported to Microsoft Excel for further analysis, and visualization was conducted using R Software version 4.3.1. Spearman's correlation coefficient and Mann-Whitney U tests were performed to determine statistical significance.

## Results

ChatGPT generated contextually relevant responses to all questions for both HF and PDA on the first attempt, that is, responses were topically appropriate and addressed the core subject of each prompt.

Ten open-ended questions related to HF were entered sequentially into ChatGPT and are presented in Table 4.

**Table 4:** Comparison of accuracy, completeness, and understandability scores for heart failure responses Data have been represented as N, mean±SD, median, or IQR IQR, interquartile range; SD, standard deviation

Question	Accuracy	Completeness	Understandability
Heart failure			
Q1. Can you explain to me what heart failure is?	6	2	77.8
Q2. What causes heart failure?	4	1	88.9
Q3. Can heart failure be cured?	5	3	77.8
Q4. How long can someone live with heart failure?	5	2	66.7
Q5. How exactly is the diagnosis made?	6	2	77.8
Q6. What is the treatment for heart failure?	5	2	72.3
Q7. What are some specific ways that a patient’s daily life would change? Can they still work, play golf, have sex, or do the laundry?	6	3	94.5
Q8. I (56, male) was diagnosed with heart failure and have been given some different drugs to use. Do I really need to use them? Can’t I just make some lifestyle changes?	6	3	100
Q9. Are there any natural remedies or nutritional supplements to help with heart failure?	5	2	94.5
Q10. I wish I didn’t have this diagnosis. What are some things that I could’ve done to prevent heart failure?	6	3	100
Mean (SD)	5.4 (0.7)	2.3 (0.7)	-
Median	5.5	2	83.35
IQR	-	-	77.8-94.5

Median accuracy score was found to be 5.5 (mean 5.4, SD 0.7), and median completeness score was found to be 2 (mean 2.3, SD 0.7). Majority of the answers generated (n=5; 50%) received the highest accuracy score, i.e., 6, while one answer (10%) received the lowest accuracy score, i.e., 4. The highest completeness score, i.e., 3 was assigned to 40% (n=4) answers, and only one answer (10%) received the lowest completeness score, i.e., 1. Understandability scores for HF had a median of 83.75% (IQR: 77.8%-94.5%).

Table 5 presents the accuracy, completeness, and understandability scores assigned to the answers generated for the 11 open-ended questions related to PDA.

**Table 5:** Comparison of accuracy, completeness, and understandability scores for PDA responses Data have been represented as N, mean±SD, median, or IQR PDA, patent ductus arteriosus; IQR, interquartile range, SD, standard deviation

Question	Accuracy	Completeness	Understandability
PDA			
Q1. Can you explain to me what PDA is?	6	2	100
Q2. What causes PDA?	5	2	100%
Q3. How is it diagnosed?	5	2	88.9
Q4. My child (3 years, female) has been diagnosed with PDA. How will it affect my child?	4	2	94.5
Q5. Is there any treatment for it?	5	3	94.5
Q6. Will my child be able to live long?	6	2	100
Q7. What activities can my child do?	5	2	87.5
Q8. What about in her adulthood? Are there any side effects that would be seen when she’s older?	4	2	100
Q9. What about if she chooses to have a child? Will she be able to have children with a PDA?	5	2	100
Q10. What are some important things to be aware of regarding her condition?	6	3	88.9
Q11. I wish she was diagnosed sooner. What symptoms should parents be aware of?	5	3	100
Mean (SD)	5 (0.7)	2.7 (0.5)	-
Median	5	2	94.5
IQR	-	-	88.9-100

Median accuracy score was found to be 5 (mean 5, SD 0.7), and median completeness score was found to be 2 (mean 2.7, SD 0.5). Of the answers generated, 27.3% (n=3) received the highest accuracy score, i.e., 6, while the majority (n=6, 54.5%) received an accuracy score of 5. The lowest accuracy score was found to be 4 and was assigned to 18.2% (n=2) answers. Majority of the answers generated (n=8, 72.7%) were scored a completeness score of 2, with only 27.3% (n=3) answers receiving a completeness score of 3. Median understandability for PDA was 94.5% (IQR: 88.9%-100%).

Table 6 presents the comparison of accuracy and completeness scores for each condition. The mean accuracy score for HF was slightly higher than for PDA (5.4 vs 5), while the mean completeness score was marginally lower (2.3 vs 2.7). However, these differences were not statistically significant as indicated by Mann-Whitney U tests (U = 41.5, p = 0.35758 for accuracy; U = 52.5, p = 0.88866 for completeness). Furthermore, Spearman’s correlation analysis showed no significant association between accuracy and completeness scores for either condition (HF: rs = 0.44854, p = 0.19353; PDA: rs = 0.21409, p = 0.52731). These findings suggest that ChatGPT performed comparably across both common and less common cardiac conditions, with no consistent link between how accurate and how complete a response was.

**Table 6:** Comparison of the mean accuracy and completeness scores for each condition, via Spearman’s R and Mann-Whitney U tests A p-value of <0.05 was considered statistically significant. PDA, patent ductus arteriosus

Condition	Heart Failure	PDA	Mann-Whitney U
Mean accuracy	5.4	5	U (accuracy) = 41.5, p = 0.35758
Mean completeness	2.3	2.7	U (completeness) = 52.5, p = 0.88866
Spearman’s R	rs = 0.44854, p (two-tailed) = 0.19353	rs = 0.21409, p (two-tailed) = 0.52731	-

## Discussion

The present study assessed the accuracy, completeness, and understandability of the answers generated by ChatGPT 3.5 in response to various medical queries regarding HF and PDA. We observed that the chatbot generated responses to all questions on the first attempt, which aligns with findings by Mediboina et al. and Rahsepar et al., who observed that ChatGPT consistently generates relevant answers to all questions, unlike chatbots such as Google Gemini in this regard [5,14].

ChatGPT demonstrated a median accuracy score of 5.5 for HF (between all correct and nearly all correct) and a score of 5 for PDA (nearly all correct). These results are comparable to Johnson et al.’s findings, which reported a median accuracy score of 5.5 across 284 questions [11]. This consistency suggests that ChatGPT maintains a similar level of accuracy across various datasets, a conclusion also supported by Cheong et al. [15]. However, the median completeness score in the present study was 2 (adequate) for both HF and PDA, compared to a score of 3 (comprehensive) in Johnson et al.’s study [11]. This discrepancy could be attributed to the larger sample size in Johnson et al.’s study and the varied difficulty levels of the questions used, which may have allowed for a more thorough evaluation of completeness.

ChatGPT also exhibits a higher understandability score for both HF and PDA, as noted similarly by studies such as those by Cheong et al. and Grimm et al. [15,16]. This can be attributed to ChatGPT’s advanced deep learning algorithms, which enable it to customize the tone of the content based on the given prompt, and this adaptability further allows ChatGPT to engage with users in a conversational manner, enhancing the clarity and understandability of the information provided [17]. However, this ease of readability may come at the expense of depth - brief or overly simplified answers may score high in understandability while lacking key clinical or contextual details, thereby lowering completeness scores [17].

The present study also observed that while the overall accuracy and completeness of answers related to HF were slightly higher compared to those for PDA, the understandability of PDA responses was notably better. This disparity is likely due to greater representation of HF topics in ChatGPT's training data, which could lead to better-informed and more detailed responses [18]. Conversely, the availability of more detailed HF information might have contributed to its lower understandability scores [19]. These observations align with those reported by Agrawal et al., who found that ChatGPT performed best in general medicine and mental health queries, likely due to the higher volume of publicly available training data [8]. A similar conclusion was reached in the study by Mehnen et al. [9].

The results of both the Spearman's correlation test and the Mann-Whitney U test suggest that the accuracy and completeness of ChatGPT's responses for HF and PDA are relatively consistent but not strongly correlated, indicating that ChatGPT has the ability to provide uniformly accurate and adequately complete information, though the depth and breadth of information might vary based on the specific condition and the nature of the questions asked [2, 4]. For HF, Spearman’s rs was 0.45, and for PDA, it was 0.21, both statistically non-significant and indicating weak to very weak positive correlations. This implies that even when ChatGPT answers are accurate, they may not necessarily be complete, suggesting an inconsistency in the depth versus correctness of its responses.

Overall, ChatGPT produced accurate content related to HF and PDA; however, some minute inaccuracies were noted, especially in terms of the PDA answers. These can be grouped into three categories for clarity:

Diagnostic omissions: for example, in the questions about “What is PDA” and “What causes PDA,” there was no mention of its occurrence in preterm infants.

Inappropriate clinical guidance: ChatGPT mentioned the use of cardiac catheterization as a diagnostic method, when it is actually a treatment approach for PDA.

Definitional errors: one instance involved the incorrect explanation of "infective endarteritis," described by ChatGPT as “an infection of the inner lining of the heart chambers and valves,” which is actually the definition of infective endocarditis. This reflects a clear conflation of two distinct conditions. Infective endarteritis refers to infection of an artery’s inner lining, often occurring at sites of abnormal blood flow such as PDA, whereas infective endocarditis involves the heart valves or endocardium.

These types of errors may reflect the limitations in ChatGPT’s clinical reasoning abilities, especially in distinguishing closely related medical conditions, and could partially explain the lack of a strong correlation between completeness and accuracy scores [5].

Limitations

Although the scoring methodology for assessing accuracy and completeness was based on previous research, it may not fully capture the complexity of ChatGPT’s responses or the nuanced clinical context in which the answers are applied. Additionally, the subjective nature of Likert-scale scoring, despite being standardized, may introduce variability and potential evaluator bias. While this study incorporated consensus scoring between two expert reviewers, formal assessment of inter-rater reliability was not performed. However, consensus was reached for all scores, which helps mitigate concerns about subjective variability. Future studies could improve robustness by including blinded third reviewers or calculating reliability metrics.

Data collection occurred at a specific point in time, potentially limiting the study's reflection of ongoing updates or advancements in ChatGPT's performance, especially since this study evaluated version 3.5, which may differ in capabilities from newer iterations such as GPT-4. This limitation affects the generalizability of the findings to current and future versions of the model.

The scale used to assess accuracy and completeness, though previously employed in similar peer-reviewed studies, has not undergone formal validation, which may affect the external validity and reproducibility of the results. Moreover, the subjective nature of the Likert scale may also impact generalizability; future studies should utilize objective scales such as the Flesch reading (FR) ease score and Flesch-Kincaid (FK) grade level [20].

The possibility of information bias must also be taken into account. ChatGPT operates on training data, and if these data are biased, it can lead to significant negative outcomes, such as reinforcing discrimination and spreading misinformation [2]. In the context of medical content, this misinformation can have severe consequences, potentially affecting patient care and health outcomes.

## Conclusions

The study found that while the accuracy and completeness of answers related to HF were slightly higher (median accuracy 5.5 vs. 5.0; median completeness 2 vs. 2.7) compared to those for PDA, the understandability of PDA responses was notably better, with a median understandability score of 94.5% versus 83.35% for HF. This suggests that ChatGPT may generate simpler, more easily understood explanations for PDA, even if those responses are less comprehensive. Several inconsistencies in the information generated, such as diagnostic inaccuracies and definitional errors, highlight ChatGPT’s potential as a patient education tool, primarily due to its generally high understandability and accessibility. However, these findings underscore the importance of continual oversight and verification of AI-generated content by medical professionals to ensure accuracy and prevent misinformation.

Future research should evaluate AI performance using larger and more diverse datasets across a broader range of medical conditions to improve generalizability. Moreover, the development of robust mechanisms for integrating medical expertise, such as expert review loops, clinical dataset fine-tuning, and standardized validation frameworks, is critical. Other objective tools such as the FR ease score and the FK grade level can also be utilized to study other aspects of AI-generated materials. These techniques could directly address observed limitations related to incomplete or inaccurate responses and enhance the reliability and clinical relevance of AI-generated patient education materials tailored for diverse patient populations.
